# Rhabdomyolysis-Induced Acute Kidney Injury in Diabetic Emergency: Underdiagnosed and an Important Association to Be Aware of

**DOI:** 10.1155/2018/4132738

**Published:** 2018-10-31

**Authors:** Ami Amin, Bhavika Gandhi, Steven Torre, Alireza Amirpour, Jennifer Cheng, Mayurkumar Patel, Mohammad A. Hossain

**Affiliations:** ^1^Department of Medicine, Internal Medicine Residency Program, Hackensack Meridian Health, Jersey Shore University Medical Center, Neptune, New Jersey 07753, USA; ^2^Endocrine Service, Nephrology Service, Hackensack Meridian Health Cancer Care, Jersey Shore University Medical Center, Neptune, New Jersey 07753, USA

## Abstract

Rhabdomyolysis is a potentially life-threatening clinical syndrome associated with muscle injury which can cause a leakage of intracellular contents, manifested from the range of being asymptomatic to a life-threatening condition causing acute kidney injury and severe electrolyte abnormalities. Rhabdomyolysis has been associated with both diabetic ketoacidosis (DKA) and hyperosmolar hyperglycemic nonketotic syndrome, though there is an increased association with rhabdomyolysis and acute kidney injury with hyperosmolar nonketonic state compared with patients with diabetic ketoacidosis. Common clinical manifestations are muscle pain, dark urine, and generalized weakness. The causes of rhabdomyolysis are broadly categorized into three groups: traumatic, nontraumatic exertional, and nontraumatic nonexertional. Here, we present a case of rhabdomyolysis-induced acute kidney injury in a patient with hyperosmolar hyperglycemic state. The patient was discharged on insulin and needed intermittent dialysis for two months. Our case highlights the importance of the rare association of rhabdomyolysis causing acute kidney injury in a diabetic emergency.

## 1. Introduction

Rhabdomyolysis is a syndrome caused by muscle breakdown which releases intracellular contents into the blood stream. Rhabdomyolysis can manifest from the range of being asymptomatic to the range of being a life-threatening condition causing acute kidney injury and severe electrolyte abnormalities. Common clinical manifestations are muscle pain, dark urine, and generalized weakness. The causes of rhabdomyolysis are broadly categorized into three groups: traumatic, nontraumatic exertional, and nontraumatic nonexertional. Here, we present a case of rhabdomyolysis-induced acute renal failure in a patient with hyperosmolar hyperglycemic state.

## 2. Case Presentation

This is a 24-year-old autistic nonverbal male who was brought into the emergency room (ER) by his mother for increasing lethargy and frequent urination for a few days. Prior to admission, he had several episodes of nonbloody vomiting, but mother denies any history of fall, prolonged immobilization, medication overdose, and increased physical activities. On admission, the patient's vitals were stable. Physical examination was significant for confusion, dehydration, and lethargy. Laboratory investigation in the emergency department revealed (Table 1) hypernatremia, highly elevated blood glucose level, elevated serum creatinine of 5.29 mg/dl (reference range 0.61–1.24 mg/dl), low bicarbonate level 13 mmol/L (reference range 24–31 mmol/L), hyperphosphatemia, and high anion gap metabolic acidosis on arterial blood gas analysis. The patient was found to be in a hyperosmolar hyperglycemic state with a serum osmolality of 395 mmol/kg (reference range 275–295 mmol/kg). Serum acetone level was large (reference range negative). Urinalysis was significant for ketones, proteinuria, high glucose level, and dark tea-colored urine. Urine blood was large; however, only few red blood cells were present. In the ER, patient was aggressively hydrated with intravenous (IV) normal saline boluses and kept on maintenance fluid with replacement of electrolytes. A computed tomography (CT) of the abdomen pelvis was consistent with pancreatitis although his amylase and lipase was normal. The patient was admitted to the intensive care unit (ICU) and was treated for DKA with standardized protocol. After resolving of acidosis, the patient was transitioned to subcutaneous insulin and started on a diabetic diet. The patient was diagnosed with diabetes type 2 on this admission. Because of new onset diabetes, glutamic acid decarboxylase was performed, which returned less then <5 IU/mL (reference range: 0.0 to 5.0, unit: IU/mL). The patient's hemoglobin A1c was elevated at 12.9% (reference range: 4.5–6.0%). The patient's acute kidney injury was attributed to dehydration with a fractional excretion of sodium (FENa) less than 1% suggestive of prerenal. His kidney function was expected to improve with volume replacement and DKA treatment. His renal function progressively worsened and became anuric and was started on hemodialysis. Because of persistent poor renal function, a creatinine phosphokinase (CPK) level was sent, which returned highly elevated at 129,940 (IU/L) (reference range 22–232 IU/L). He was maintained on a high rate of intravenous normal saline and hemodialysis. In the next two days, his CPK level started to trend downward and became normal upon on discharge. The patient required 2 months intermittent hemodialysis and developed chronic kidney disease stage 2 with a glomerular filtration rate of 80.

## 3. Discussion

Hyperosmolar state is an extremely rare cause of rhabdomyolysis. The causes of rhabdomyolysis are broadly categorized into three groups: traumatic, nontraumatic exertional, and nontraumatic nonexertional. Out of the three categories of rhabdomyolysis, our case belongs to the group of nontraumatic, nonexertional type. There has been a link associating diabetic emergencies and rhabdomyolysis [1, 2] and increased mortality rate. The link between rhabdomyolysis and diabetic emergencies is not well known, and that is why there is a potential to miss of the diagnosis of rhabdomyolysis.

In a study titled “Rhabdomyolysis in Diabetic Emergencies,” there were 44 cases of rhabdomyolysis out of 265 patients. The study showed that subclinical rhabdomyolysis in diabetic emergencies did occur more frequently than assumed [3, 4]. With minimal symptoms and clinical manifestations, the diagnosis can be missed. Our patient presented with the clinical manifestation of kidney failure. However, we had the additional challenge of our patient being mostly nonverbal, unable to express his complaints. This patient presented with new onset diabetes and hyperosmolar hyperglycemic state with severe dehydration. Initially, AKI was thought to be caused by dehydration due to hyperosmolar hyperglycemic state, and CPK was not checked initially. It is a great reminder for the clinician not to be limited to a single diagnosis and needs to consider other potential causes of acute kidney injury in diabetic emergencies when renal function did not improve in spite of having hemodialysis.

Even though rhabdomyolysis has been associated with both DKA and hyperosmolar hyperglycemic nonketotic syndrome, there is an increased association with rhabdomyolysis and acute kidney injury with hyperosmolar nonketonic state compared with patient with diabetic ketoacidosis. Although the exact mechanism of action of a hyperosmolar state causing rhabdomyolysis remains unclear, there have been theories postulating the etiology [5]. The high osmolarity can potentially cause the breakdown of the muscle cell wall [5, 6]. Also electrolyte abnormalities such as hypernatremia, hypokalemia, and hypophosphatemia seen in hyperosmolar hyperglycemia state also contribute to rhabdomyolysis [4, 7]. With intravenous insulin infusion in diabetic emergencies, there is a rapid shift of electrolytes from extracellular to intracellular. With insulin, there is a decrease in serum potassium and serum phosphate. Hypokalemia itself is a known metabolic cause of rhabdomyolysis [8].

In most cases of diabetic emergency, acute kidney injury usually presumed to be due to dehydration and diabetes itself, and rhabdomyolysis remained underdiagnosed, which imposes potential consequences and increases mortality. Delayed diagnosis and treatment of rhabdomyolysis may lead to irreversible kidney damage progress to chronic kidney disease. With the patient's history of autism, there was a missed opportunity for screening for an underlying myopathy, which could be revealed in acute transient rhabdomyolysis [9]. As a clinician, we should be more vigilant to look for this association and can prevent lifelong complications of renal failure.

## 4. Conclusion

Due to the high mortality rates associated with rhabdomyolysis and hyperosmolar hyperglycemic nonketotic syndrome, it is important to establish an early diagnosis. Routine screening by ordering CPK levels should be considered in patients admitted with diabetic emergencies. Acute kidney injury associated with rhabdomyolysis plays a role in the overall outcome of mortality. Rhabdomyolysis should be suspected in patients presenting with diabetic emergencies and acute kidney injury when common causes have been excluded and treated accordingly without improvement of kidney function.

## Figures and Tables

**Table 1:** Laboratory findings.

Lab	Patient	Reference range
Sodium (mmol/l)	146	136–145
Potassium (mmol/l)	4.4	3.5–5.2
Chloride (mmol/l)	106	96–110
Carbon dioxide (mmol/l)	13	24–31
Anion gap (mEq/L)	27	8–16
Glucose (mg/dl)	1572	70–110
Serum urea nitrogen (mg/dl)	76	5–25
Creatinine (mg/dl)	5.29	0.61–1.24
Calculated osmolality (mosm/kg)	395	275–295
Phosphorous (mg/dl)	6.3	2.5–4.6
Calcium (mg/dl)	10.5	8.5–10.5
Albumin (g/dl)	4.3	3.5–5
LDH ((IU)/L)	6320	91–200
Creatinine phosphokinase ((IU)/L)	129,440	22–232

Arterial blood gas		
pH	7.171	7.35–7.45
pCO_2_ (mmHg)	35.6	35–50
pO_2_ (mmHg)	49.8	85–106
O_2_ saturation	70.8	95–98
Bicarbonate (mmol/L)	12.5	22–26
Hemoglobin A1c (%)	12.9	4.5–6%

Urinalysis		
Urine glucose (mg/dl)	>1000	Negative
Urine blood	*Large*	*Negative*
Urine RBC	3–5	Negative
Urine ketones (mg/dl)	15	Negative
Urine protein (mg/dl)	100	Negative
